# Dissection of the Inflammatory Bowel Disease Transcriptome Using Genome-Wide cDNA Microarrays

**DOI:** 10.1371/journal.pmed.0020199

**Published:** 2005-08-23

**Authors:** Christine M Costello, Nancy Mah, Robert Häsler, Philip Rosenstiel, Georg H Waetzig, Andreas Hahn, Tim Lu, Yesim Gurbuz, Susanna Nikolaus, Mario Albrecht, Jochen Hampe, Ralph Lucius, Günther Klöppel, Holger Eickhoff, Hans Lehrach, Thomas Lengauer, Stefan Schreiber

**Affiliations:** 1Institute for Clinical Molecular Biology, University Hospital Schleswig Holstein, Christian-Albrechts University of Kiel, Kiel, Germany,; **2**CONARIS Research Institute, Kiel, Germany,; **3**Department of Computational Biology and Applied Algorithmics, Max-Planck-Institute for Informatics, Saarbrücken, Germany,; **4**Institute of Pathology, University Hospital Schleswig Holstein, Kiel, Germany,; **5**Department of General Internal Medicine, University Hospital Schleswig Holstein, Kiel, Germany,; **6**Institute of Anatomy, University Hospital Schleswig Holstein, Kiel, Germany,; **7**Max-Planck-Institute for Molecular Genetics, Berlin, Germany; Rikshospitalet University of OsloNorway

## Abstract

**Background:**

The differential pathophysiologic mechanisms that trigger and maintain the two forms of inflammatory bowel disease (IBD), Crohn disease (CD), and ulcerative colitis (UC) are only partially understood. cDNA microarrays can be used to decipher gene regulation events at a genome-wide level and to identify novel unknown genes that might be involved in perpetuating inflammatory disease progression.

**Methods and Findings:**

High-density cDNA microarrays representing 33,792 UniGene clusters were prepared. Biopsies were taken from the sigmoid colon of normal controls (*n* = 11), CD patients (*n* = 10) and UC patients (*n* = 10). ^33^P-radiolabeled cDNA from purified poly(A)^+^ RNA extracted from biopsies (unpooled) was hybridized to the arrays. We identified 500 and 272 transcripts differentially regulated in CD and UC, respectively. Interesting hits were independently verified by real-time PCR in a second sample of 100 individuals, and immunohistochemistry was used for exemplary localization. The main findings point to novel molecules important in abnormal immune regulation and the highly disturbed cell biology of colonic epithelial cells in IBD pathogenesis, e.g., *CYLD (cylindromatosis, turban tumor syndrome)* and *CDH11 (cadherin 11, type 2).* By the nature of the array setup, many of the genes identified were to our knowledge previously uncharacterized, and prediction of the putative function of a subsection of these genes indicate that some could be involved in early events in disease pathophysiology.

**Conclusion:**

A comprehensive set of candidate genes not previously associated with IBD was revealed, which underlines the polygenic and complex nature of the disease. It points out substantial differences in pathophysiology between CD and UC. The multiple unknown genes identified may stimulate new research in the fields of barrier mechanisms and cell signalling in the context of IBD, and ultimately new therapeutic approaches.

## Abstract 2

cDNA analysis of colonic samples from patients with Crohn disease and ulcerative colitis suggests substantial differences in pathophysiology between the two diseases.

## Introduction

The two main forms of inflammatory bowel disease (IBD), Crohn disease (CD) and ulcerative colitis (UC), are both characterised by an aberrant immune response of the intestinal mucosa. The current understanding of disease pathogenesis suggests a complex interplay of multiple environmental and genetic factors [1]. Although clinical, endoscopic, histopathologic, and radiologic criteria exist to distinguish CD from UC, considerable overlap is found in clinical criteria, understanding of pathophysiology, and therapy [2]. The enormous complexity of pathophysiology mandates a systematic approach to identify the molecular events that cause and perpetuate these chronic, relapsing inflammatory disorders.

In the search for genes that cause IBD, several genetic linkage studies, which identify the approximate chromosomal locations of disease susceptibility genes, have been carried out [3]. This technique, in combination with the more classical candidate gene approach, led to the identification of the first disease-associated variants in the *NOD2/CARD15* gene on chromosome 16 [4–6]. More recently, we have identified *discs, large homolog 5* *(DLG5; Drosophila)* on chromosome 10q23, which encodes a scaffolding protein potentially involved in the maintenance of epithelial integrity, as an IBD susceptibility gene [7]. Concomitantly, functional variants in the *solute carrier family 22 (organic cation transporter)* genes, *SLC22A4* and *5* on chromosome 5q31, were found to be associated with CD [8]. The high number of linkage regions in IBD and the multiplicity of association findings suggest enormous complexity behind the polygenic risk between patients. Genetic susceptibility factors are therefore unlikely to serve as molecular targets for direct therapeutic application. Key molecules in pathophysiology, downstream of points of convergence between the chains of regulatory events originating from different etiologic factors, are more likely targets for successful therapeutic interventions. This can be illustrated with the example of *tumour necrosis factor-alpha (TNF-α)*, a molecule that does not appear to be causative in CD, but allows effective interruption of the inflammatory cascades through administration of a neutralizing, recombinant antibody construct [9]. The example of *TNF-α* also illustrates that a successful therapeutic approach may not target disease-specific pathophysiology, but rather molecules that are of general importance for inflammation pathophysiology, and therefore involved in a host of inflammatory disorders.

The sequencing of the human genome and the concurrent establishment of the expressed sequence tag (EST) clone database [10] have greatly improved the possibility of finding new pathophysiology-relevant genes. One technique now adopted in this latter endeavour is microarray technology, where the transcripts of thousands of genes can be simultaneously investigated. Three exploratory microarray studies carried out on intestinal mucosa samples to date broadly concur on the known genes found to be associated with IBD [11–13].

In the present study, we have used mucosal biopsies obtained by endoscopy, and not isolated cell populations, because IBD represents the rare case of a nonmalignant human disorder in which relevant disease tissue can be obtained without surgery and without any technical variance introduced by a cell isolation process. We set up a system for expression profiling using PCR-amplified cDNA clone inserts from a large whole genome collection derived from clustered EST libraries spotted on nylon filters. This genome-wide gene set was optimized for clones representing clusters from unknown ESTs and genes. Due to the clustering algorithm used, some well-known genes are therefore not represented. Preliminary experiments have demonstrated a limited overlap with the genome-wide arrays from Affymetrix with reproducible but different findings generated on both systems [14]. The two-stage design of the present experiment used a sensitive radioactive detection method for the array signals (resulting in a lower dynamic range than Affymetrix arrays) followed by real-time PCR (TaqMan) for a quantitative assessment of the changes detected. In carrying out this study, we aimed to broaden our understanding of gene regulation events in CD and UC, and identify novel genes involved in perpetuating inflammatory disease progression.

## Methods

### Patients

#### Patient group 1

This group was composed of all samples (representing 31 individuals, recruited between 1999 and 2003) analysed by cDNA microarrays (Table 1). Eleven individuals (five females [F]; mean age 51.4 y; range 25–84 y) were included in the study as the normal population, with endoscopic and histological examination yielding no significant pathological findings. Indications for colonoscopy in this group included colonic cancer surveillance and previous nonspecific changes in stool habits. To investigate gene expression in IBD, ten patients with active CD (4F; mean age 33.6 y; range 19–66 y) and ten patients with active UC (2F; mean age 34.8 y; range 27–51 y) were sequentially recruited. All endoscopic biopsies were taken from a defined area of the sigmoid colon (at 20–30 cm measured during withdrawal), and immediately snap-frozen in liquid nitrogen. A second set of biopsies was taken from the same region for histological scoring. Clinical disease activity was documented using established clinical parameters of the Crohn disease activity index (CDAI) in CD [15] and the colitis activity index (CAI) [16,17] for UC patients. Inclusion criteria were clinically (CDAI > 150 or CAI ≥ 4) and endoscopically active disease in the sigmoid colon at the time of sampling. Patients also had to be free of all medication used for the treatment of IBD (other than low-dose 5-aminosalicylic acid [5-ASA]) for a minimum of 6 wk prior to endoscopy. More than 300 patients were screened to recruit the study population.

**Table 1:**
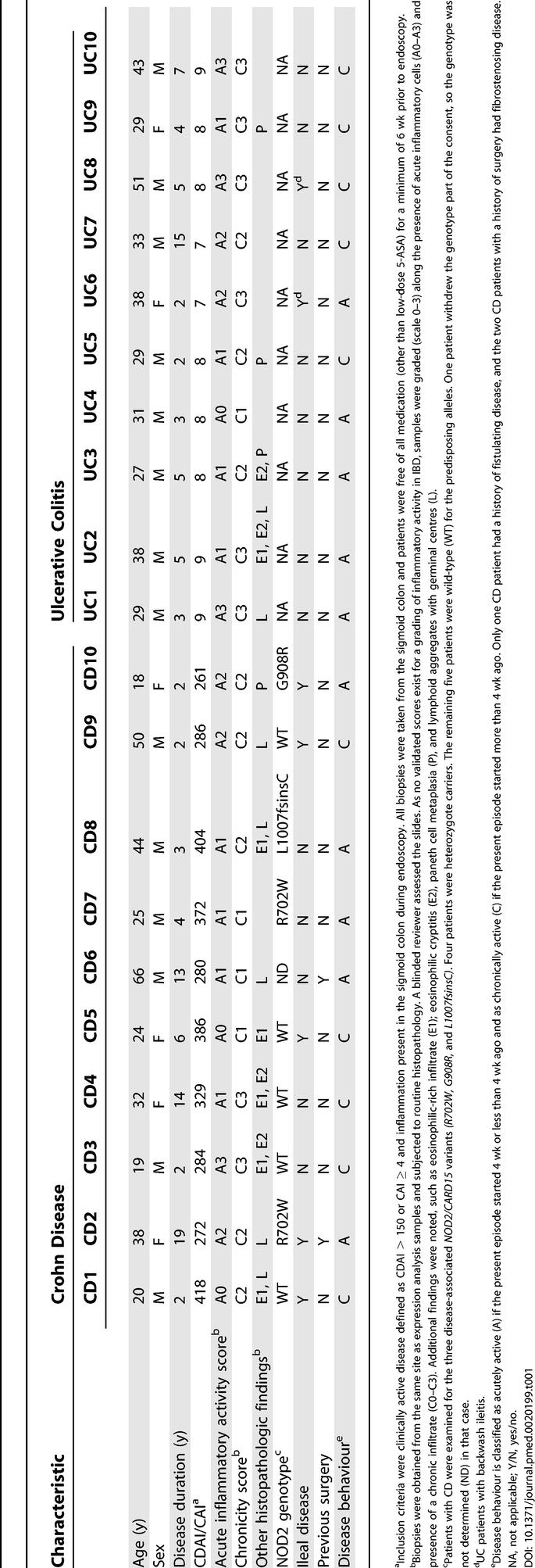
Clinical Characteristics of the IBD Patient Group 1

#### Patient group 2

To verify and investigate the molecular epidemiology of selected signals seen in genome-wide array experiments, patient group 1 was extended from 31 to 100 individuals and analysed by quantitative real-time PCR (Table 2). IBD patients used in group 2 consisted of 27 CD patients (17F; mean age 32.1 y; range 17–45 y) and 35 UC patients (20F; mean age 33.8 y; range 18–74 y). The patients were recruited between 1999 and 2004 and were selected using similar criteria as outlined above, i.e., all patients had active inflammation in the colon and were clinically active (CDAI > 150 or CAI ≥ 4). Patients were allowed 5-ASA or glucocorticoids, but not immunosuppressants or biologicals. The normal control population consisted of 21 individuals (11F; mean age 48.9 y; range 16–84 y), who had no significant pathological findings following endoscopic examination for changes in stool habits, abdominal pain, or upper gastrointestinal bleeding or cancer surveillance. As disease specificity controls, 17 patients (9F; mean age 56 y; range 17–80 y) with colonic disease but not IBD, were also included (Table 2). These disease specificity controls (DCs) included nine non-inflamed and eight inflamed biopsies samples from patients with infectious diarrhoea, other forms of gastrointestinal inflammation, or irritable bowel syndrome. No restriction on medications was specified in the inclusion criteria for DCs and all concomitant medications are listed in Table 2. A minimum of 19 normal controls, 16 UC, 19 CD, and 14 DC samples were used for each real-time PCR assay, depending on the availability of the patient samples and the plate layout at the time the real-time cDNA plates were produced.

**Table 2:**
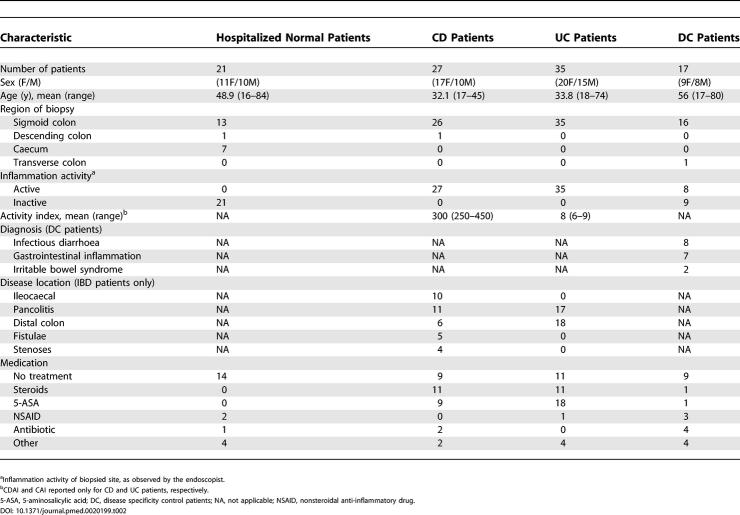
Characteristics of Patient Group 2, Including DCs

Biopsies from all patients in the microarray study were taken from the sigmoid colon. For the real-time PCR work, biopsies were also taken from other regions of the large intestine (Table 2). We conducted extensive preparatory studies in which we systematically compared the influence of anatomical region on the expression profiles obtained. Significant differences in expression patterns between the sigmoid colon, descending colon, and transverse colon/caecum were not observed (unpublished data). Additionally, animal data has shown that different regions of the GI tract (stomach, small intestine, and colon), but not regions within the colon, were distinguishable by expression profiles [18]. We therefore considered regions within the large bowel to be equivalent and that sampling from different areas of the large bowel would have negligible effect on differential gene expression.

### Informed Consent and Approval by the Ethics Committee

All patients included in this study consented to additional research biopsies being taken 24 h prior to endoscopy. One patient withdrew the genotype part of his consent. The procedures in the study protocol were approved by the Ethic Committee of the Medical Faculty of the Christian-Albrechts-University prior to the start of the study.

### Target Preparation and Hybridisation to cDNA Microarrays

cDNA clone inserts from 88 microtiter plates containing 384 wells (i.e., 33,792 inserts total) from the Human UniGene set RZPD 1 clone set (German Resource Center for Genome Research [RZPD], Berlin, Germany; http://www.rzpd.de/) were amplified by PCR and spotted onto nylon filters [14]. Since the redundancy of this clone set was previously estimated to be approximately 1.44-fold [19], the 33,792 clones on the cDNA microarray represent approximately 23,000 unique transcripts. Total RNA was isolated from snap-frozen biopsies, and mRNA isolation, radioactive labelling of mRNA, and subsequent steps (hybridisation of the radiolabelled target, washing, and scanning of filters) were carried out as previously described [14].

### Data Analysis

Image gridding and spot quantitation were carried out using VisualGrid software (GPC Biotech, Martinsried, Germany; http://www.gpc-biotech.com, and results were imported into a custom-made database for further analysis. The ranked expression data distribution was found to follow Zipf's law, and the log-transformed datasets were normalized following this principle [20,21]. Any hybridisation signals that had a value that fell below two standard deviations of the mean background were considered as indistinguishable from background and were set to zero. In the few cases where duplicate expression values for the same genes were not similar (outliers or false positives), all readings for this gene were eliminated. The entire microarray dataset was not normally distributed and, therefore, in order not to assume a specific distribution of data, significantly differentially regulated genes between conditions were identified by a nonparametric test (Mann-Whitney U test). Fold-changes were calculated based on the median of each experimental group.

In order to identify potential gene function, the SOURCE database (http://source.stanford.edu) was used to retrieve Gene Ontology terms, after genes expressed more than once were removed (Dataset S1). Systematic analysis of functional groups in microarray datasets is a complex issue, which is still under discussion. We used the published method of Tavazoie et al. [22] to support the validity of the three functional groups we selected for discussion. Briefly, this method determines whether the selection of a subset of genes results in over-representation/enrichment of functional groups, compared to the genes in these functional groups on the entire array. We compared our gene findings with those from three other smaller IBD studies [11–13] to determine if the proposed disease mechanisms were similar.

### Quality Controls and Assessment of Technical Variance

In order to ascertain data reliability and reproducibility, the expression levels of each of the 33,792 clones were measured in duplicate in each experiment. The resulting correlation coefficient was very high (*r* = 0.99), indicating that hybridisation signals were highly reproducible (unpublished data). To assess microarray and hybridisation-based experimental variability, hybridisation was performed on five separate microarrays using the same RNA sample (extracted from an intestinal surgical specimen; unpublished data). We performed pairwise comparisons between all possible combinations of filters. We computed the fold-change observed for each of the 33,792 replicate signals, which should theoretically be 1 (i.e., no fold-change) in all cases, but varies due to experimental noise. We recorded the fold-change observed at the 95th percentile of the distribution for each pairwise comparison and took the average of these values. This provided us with a fold-change of 1.2 as the significance threshold to claim differential expression. This threshold of positive gene identification is lower than reported for many studies; i.e., array studies using Affymetrix arrays usually consider a 2-fold or greater change between two conditions as significant. It is worth noting that common fluorescently labelled oligonucleotide-based array systems (i.e., Affymetrix) amplify the signal with biotin-streptavidin complexes, which is not the case with radioactive detection, hence the fold-change cutoff criteria are not comparable between these two platforms. However, we substantiated our threshold findings by carrying out additional statistical analysis with an additional well-established microarray analysis package. Using significance analysis of microarrays (SAM) software (set to a maximum false discovery rate of 4%) to retest our analysis based on fold-changes, 10% of our significantly regulated genes were rejected by SAM. This difference comes mainly from genes that have barely passed the significance level of our own analysis (unpublished data). Heat maps of differentially regulated genes were generated using Spotfire Decision site 8.1 software (Spotfire, Somerville, Massachusetts, United States; http://www.spotfire.com/).

### Verification of Clone Sequences

Differentially expressed clones were bidirectionally sequenced from PCR products of clone inserts or bacterial plasmids as previously described [14]. Contigs were generated if the clone ends overlapped (Sequencher software; Gene Codes Corporation, Ann Arbor, Michigan, United States) and identified by BLAST searching (http://www.ncbi.nlm.nih.gov/BLAST/). All genes reported in this study have been sequence-verified in this manner. Incorrect clone annotations were found in 27% of cases. This annotation error rate is similar to that reported for IMAGE clone sets in other studies [23,24].

### Independent Quantitation of Microarray Results by TaqMan PCR

Selected gene expression signals were independently quantitated with real-time PCR (TaqMan) on patient group 2. Probes and primers were either designed to non-redundant sequences using Primer Express V2.0 (Applied Biosystems, Foster City, California. United States), or ordered from Applied Biosystems as Assays-on-Demand Gene Expression Assays (Table S1). Total RNA (1 μg) was reverse-transcribed to cDNA according to the manufacturer's instructions (MultiScribe Reverse Transcriptase, Applied Biosystems). Reactions were carried out on the ABI PRISM Sequence 7700 Detection System (Applied Biosystems) and relative transcript levels were determined using β-actin as the endogenous control gene. Statistically significant differences between control and IBD samples were determined using a Mann-Whitney U test. A *p-*value of less than 0.05 was considered significant.

### Histological Rating of Disease Activity

A single pathologist (Y. G.), blinded to disease status and the experimental results, performed the histological rating of inflammatory activity in biopsy samples taken from the same anatomical location as the samples used for microarray experiments. The normal group showed no pathologic signs of activity in the histological examination (unpublished data). As detailed in Table 1, acute inflammatory activity and chronic changes were rated, with additional endoscopic findings also noted.

### Immunohistochemistry

In order to determine protein localization, paraffin-embedded biopsies from normal controls (*n* = 5) and from patients with CD (*n* = 5) and UC (*n* = 6), which were obtained in parallel from the same sites as the biopsies used for the expression analysis studies, were analysed. This procedure is described in more detail elsewhere [25]. Briefly, 7-μm sections were subjected to heat-induced antigen retrieval in 0.01 M EDTA solution (pH 8) for 10 min. After blocking in a solution of 0.75% BSA in PBS for 20 min, the sections were washed three times in PBS and incubated for 1 h with the respective primary antibodies (monoclonal carcinoembryonic antigen-related cell adhesion molecule 1 [CEACAM1] 4D1/C2, protein kinase C beta 1 [PRKCB1; Pharmingen, San Diego, California, United States], and casein kinase 1, delta [[CSNK1D; Santa Cruz Biotechnology, Santa Cruz, California, United States]. After washing in PBS (three times for 10 min), the sections were incubated with peroxidase-conjugated rabbit anti-mouse secondary antibody (Sigma, Deisenhofen, Germany; 1:100) for 30 min, washed, and stained with goat anti-rabbit IgG (Sigma; 1:200, 30 min), before processing with diaminobenzidine and embedding in Aquatek (Merck, Hawthorne, New York, United States). For specificity controls, (i) the primary antibodies were omitted and (ii) normal sera were used from the species in which the primary antibodies were raised. No specific staining could be detected using either of these combinations (unpublished data).

### Prediction of Function in Unknown Genes

In order to identify novel genes, unknown differentially regulated clones were examined in more detail. cDNA clone sequences were searched against the GenBank nonredundant database by means of BLAST searching. The best BLAST hit (sequence identity close to 100%) was used to extend the sequences to longer mRNA transcripts. InterPro (http://www.ebi.ac.uk/InterProScan, SMART (http://smart.embl-heidelberg.de) and Pfam (http://www.sanger.ac.uk/Software/Pfam) search methods (with standard parameter sets) were used to detect known protein domains and functional sequence motifs in putative open reading frames (ORFs) longer than 100 amino acids. Close homologies of the ORFs were found through their association to UniGene clusters in humans and other species. In cases of ORFs without a detectable homology to known genes, these transcripts were mapped to genomic locations by BLAST searching and examined in terms of their genomic context. Additionally, we used a previously published method for prediction of function of mouse transcripts [26], and applied a similarity analysis in order to predict the function based on common regulation patterns between known functional groups and unknown transcripts.

## Results

### Expression Profiles of the IBD Colonic Mucosa

**Figure 1:**
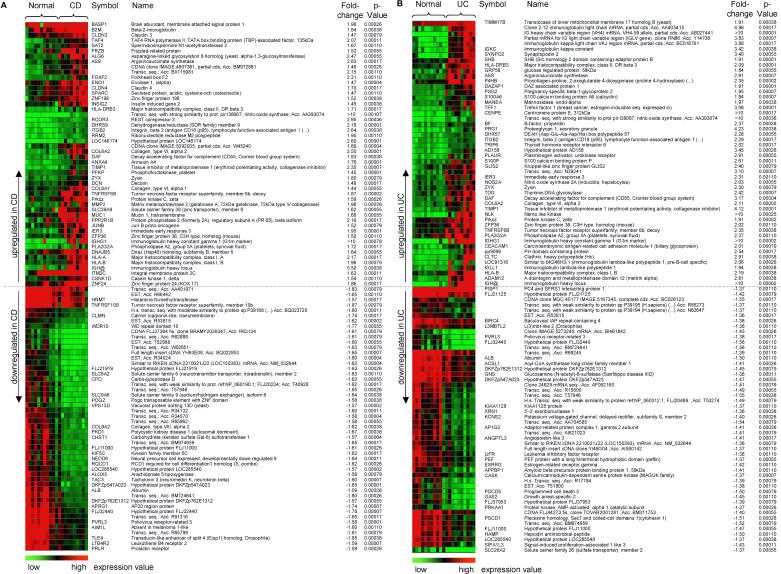
Heat Map of Differentially Expressed Genes in Normal Controls Compared to IBD (A) The top 40 up-regulated and the top 40 down-regulated between normal controls (*n* = 11) and CD patients (*n* = 10). (B) The top 40 up-regulated and the top 40 down-regulated genes between normal controls (n = 11) and UC patients (*n* = 10). Selection criteria were *p* ≤ 0.0015 (based on Mann-Whitney U test) and a fold-change of 1.2 or greater. All genes presented in this heat map were sequence verified.

Samples from sigmoid colon that were taken from normal controls (*n* = 11), patients with CD (*n* = 10), and patients with UC (*n* = 10) were used to compare differences between normal and inflamed intestinal mucosa. After implementing established analysis criteria, 650 genes were found to be differentially regulated between normal controls and at least one of the IBD subtypes. Of these 650 genes, 500 transcripts (81 up-regulated) were identified as differentially regulated between normal controls and CD patients. Two hundred and seventy-two differentially regulated transcripts (157 up-regulated) were identified between normal controls and UC patients. At our cutoff level, 122 genes were dysregulated in both diseases, and approximately 44% and 36% of all differentially regulated genes represented unknown genes in CD and UC, respectively. Heat maps shown in Figure 1 represent the top 40 up- and down-regulated genes for each experimental group. The complete list of all differentially regulated genes identified in this study (including reference to chromosomal areas previously implicated in IBD linkage studies) can be found in Table S2.

In order to delineate the molecular fingerprint of each of the two disease subtypes, the differentially regulated genes were assigned to functional groups based on classification by Gene Ontology (http://www.geneontology.org/) and references in the literature. The majority of genes fell into the following groups, which are listed as such in Table 3: immune and inflammatory responses; oncogenesis, cell proliferation, and growth; and structure and permeability. The significance of this observation was tested according to Tavazoie et al. [22]. The *p*-value for the overall significance for an over-representation of genes in the category “immune and inflammatory responses” was 0.00062. This enrichment was mainly driven by genes that were up-regulated in CD (*p* = 0.0011) and UC (*p* = 0.0026). An enrichment of genes associated with cell growth and proliferation (*p* = 0.0404) was driven by genes that were up-regulated in UC, while genes associated with structure and permeability (*p* = 0.0309) are mostly found in the category of genes up-regulated in CD.

**Table 3:**
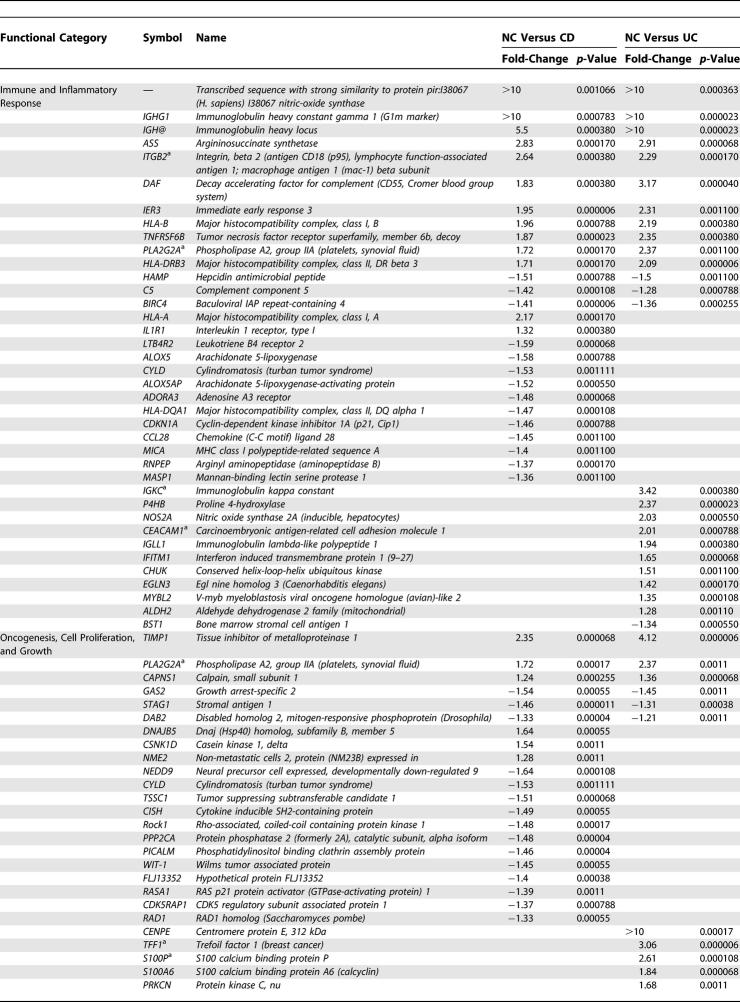
Functional Groups of Differentially Regulated Genes

**Table 3:**
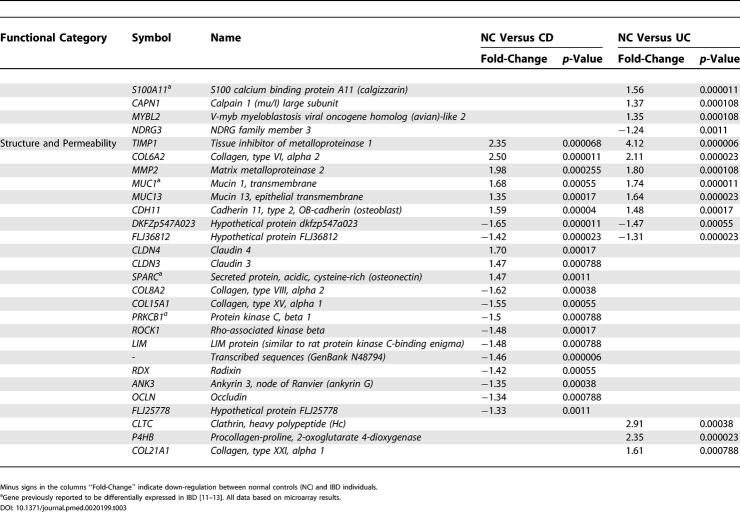
Continued

### Functional Prediction of Unknown Genes

To decipher potential roles for unknown genes, we further analysed some of the top differentially expressed unknown genes between normal controls and IBD (Table 4). Some of the transcripts had no known sequence homologies (e.g., GenBank accession number BC006384), whereas others were in UniGene clusters with sequences homologous to structural/cytoskeletal proteins (e.g., AK022544). Additionally, some transcripts showed sequence homology to cell adhesion genes (e.g., N48794 and AF087994). The gene represented by GenBank accession number AL117511 may be involved in vacuolar sorting, and AK056932 is a putative transmembrane phosphate acyltransferase. Additional gene products contain DNA-binding domains and may function as transcription factors (e.g., N39296 and AW953679), or represent a possible kinase (e.g., AB067499) or may play a role in intracellular signalling (e.g., BC008744). Of the unknown genes analysed, 90% have homologues in other species, lending credibility to the hypothesis that these as yet uncharacterised genes must have an important function since they are conserved in the evolutionary process. Additionally, predictions based on similarities of regulation patterns of known functional groups are listed in Table 4.

**Table 4:**
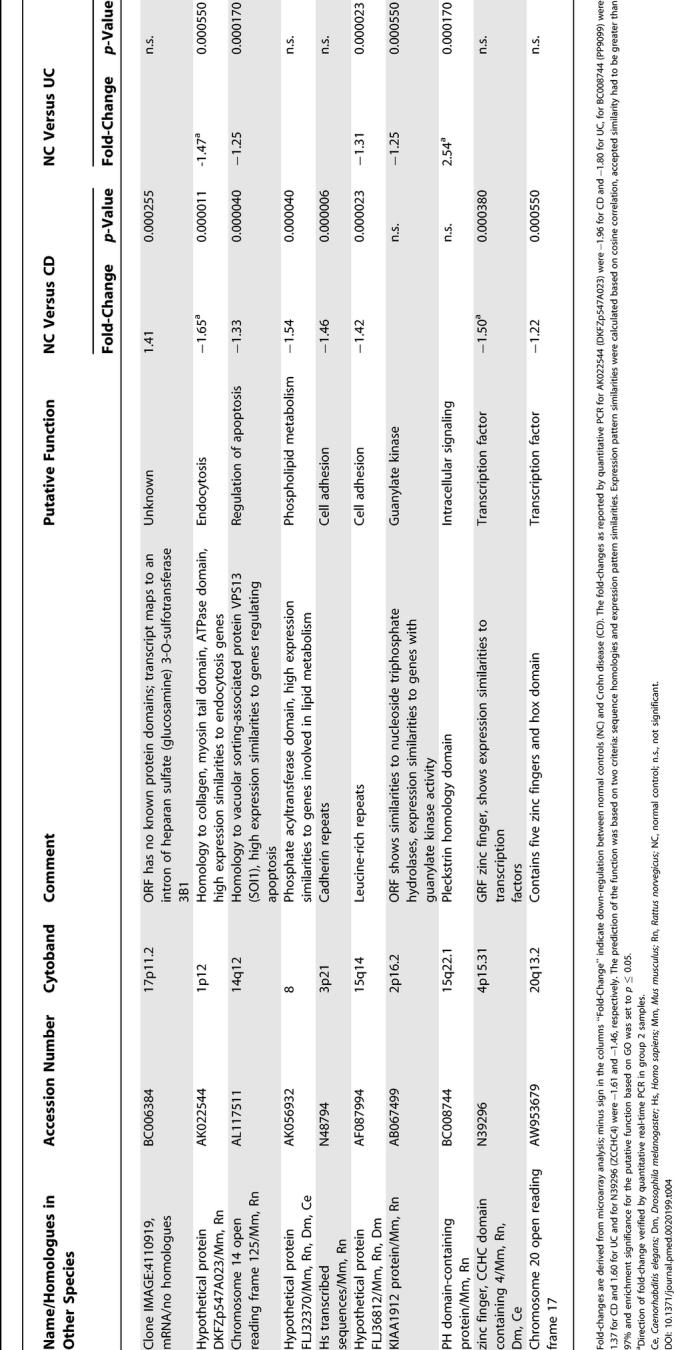
In Silico (InterPro, SMART and Pfam Search Methods) Analysis of Top Differentially Expressed Unknown Genes between Normal Controls and CD or UC, Respectively

### Independent Quantification by Fluorescent Real-Time PCR

**Figure 2:**
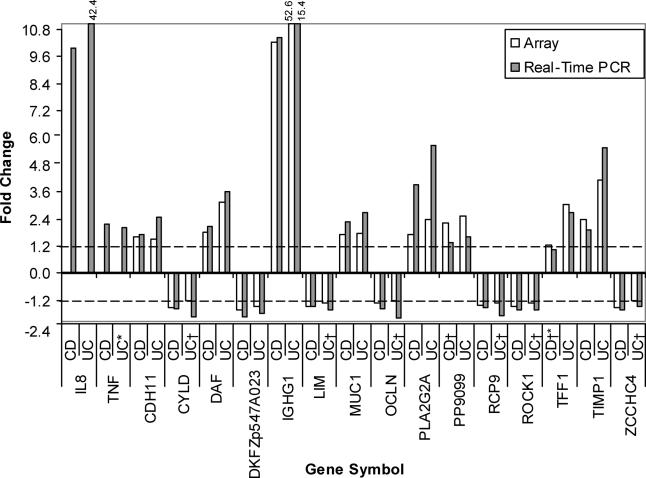
Microarray Results and Corresponding Quantitative Real-Time PCR for Differentially Regulated Genes in CD or UC Compared to Normal Controls Genes were chosen on the basis of their dysregulation in IBD and represent both known genes from functional groups discussed and genes of unknown function. Quantitative real-time PCR was carried out on individual samples from group 2 patients (14–18 normal controls, 19–33 UC, and 17–22 CD samples, depending on the availability of the patient samples at the time the plates were produced), except for IL-8 and TNF-α (not on array), which were tested in group 1 patients (11 normal controls, ten UC, and ten CD patient samples) as a proof-of-principle measure. The extended cohort of group 2 patients includes those with active disease and using anti-inflammatory drugs (but not immunosuppressants or biologicals), whereas group 1 patients had active disease and were medication-free for 6 wk. Results are summarised by a ratio of medians (CD:normal or UC:normal). Complete results, including box-plots and number of samples analysed in each assay, are included in Figure S1. All results were significantly differentially regulated except for marked results; single dagger indicates that array result was not significant (*p* > 0.0015 or fold-change < 1.2); asterisk indicates that real-time PCR result was not significant (*p* > 0.05). A dashed line represents the fold-change level of 1.2.

Real-time TaqMan PCR on selected transcripts was carried out as a sensitive independent verification method of the microarray results (Figure 2). The population of 31 patients used for microarray studies (group 1) was extended for the real-time PCR experiments to 100 patients (group 2). Group 1 patients did not exhibit significant differences from group 2 patients on the level of gene expression, as verified by groupwise comparison. Genes for real-time PCR were chosen based upon their dysregulation in IBD, and representation within the three main functional groups discussed later. Differentially regulated transcripts of unknown function were also included. As an additional control for the suitability of the samples and the stability of the overall approach, *TNF-α* and *interleukin-8 (IL-8)* were used, as small soluble cytokine mediators not present on the array system but known as hallmarks of IBD pathophysiology. As expected, overexpression of *IL-8* in both CD and UC and *TNF-α* in CD was observed in the samples used. An additional 15 genes on the microarrays were then analysed in the extended real-time patient cohort (i.e., patient group 2). The 15 real-time PCR experiments that were carried out are summarised in Figures S1 and 2 and Table 5. Genes that were significantly up-regulated in both microarray and real-time PCR in the two IBD subtypes (compared to normal controls), include *cadherin-11 (CDH11), decay accelerating factor for complement (DAF), immunoglobulin heavy constant gamma 1 (IGHG1), mucin 1 (MUC1), phospholipase A2, group IIA (PLA2G2A),* and *tissue inhibitor of metalloproteinase 1 (TIMP1)*. The unknown gene, *DKFZp547A023* (GenBank accession number AK022544; see Table 4), was confirmed by real-time PCR to be down-regulated in both disease groups. In the case of *cylindromatosis (CYLD),* *calcitonin gene-related peptide-receptor component protein (RCP9), LIM protein (LIM), occludin (OCLN), Rho-associated, coiled-coil containing protein kinase 1 (ROCK1),* and *zinc finger, CCHC domain containing 4 (ZCCHC4),* the microarray results showed that these genes were down-regulated in both IBD subtypes, but failed to reach significance in the UC cohort. However, results from the extended real-time analysis showed that these six genes were significantly down-regulated in both IBD subtypes. *PH domain-containing protein* (*PP9099,* BC008744; see Table 4) was up-regulated in both diseases on the microarray, but our microarray cutoff criteria were not reached in the CD population. Real-time analysis on the extended IBD population demonstrated this gene was significantly up-regulated in both IBD subtypes. One gene, *trefoil factor 1 (TFF1),* was found to be up-regulated in UC on the microarray but failed to reach significance for CD, a result that was confirmed by real-time PCR in the extended group 2 samples. These results strongly emphasize the importance of using a high-throughput quantitative technique to follow positive signals from microarray experiments into larger and diverse patient populations.

**Table 5:**
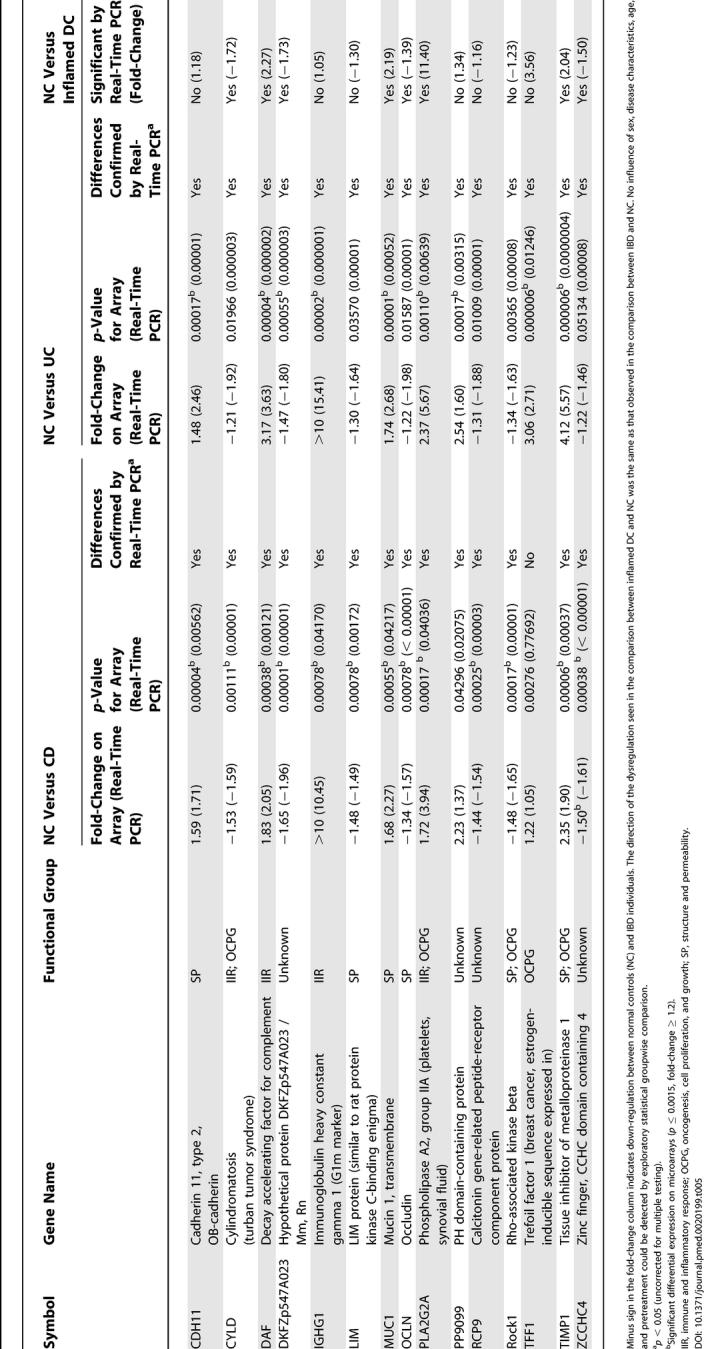
Summary of Array and Real-Time PCR Results

### Expression Profiles in Non-IBD Disease Samples

Disease specificity is an important issue in the detection of differential gene expression between IBD and normal controls. Real-time PCR was carried out on patients that had colonic disease (DC, *n* =17), but not IBD (Figures S1 and 3). These DC samples were again divided into noninflamed and inflamed conditions. No significant difference was observed between the expression of any of the 15 genes tested between normal controls and noninflamed disease specificity controls. Eight genes (*CYLD, DAF, DKFZp547A023, MUC1, OCLN, PLA2G2A, TIMP1,* and *ZCCHC4;* Table 5) were significantly differentially regulated between normal controls and inflamed DC, and the direction of change was the same as that observed in IBD. This observation suggests that these eight gene findings probably reflect general inflammation pathophysiology rather than events specific for IBD. However, unlike in the IBD cohort, the expression of the remaining seven genes (*CDH11, IGHG1, PP9099, TFF1, LIM, ROCK,* and *RCP9;* Table 5) was not significantly different in between normal controls and the inflamed DC group.

**Figure 3:**
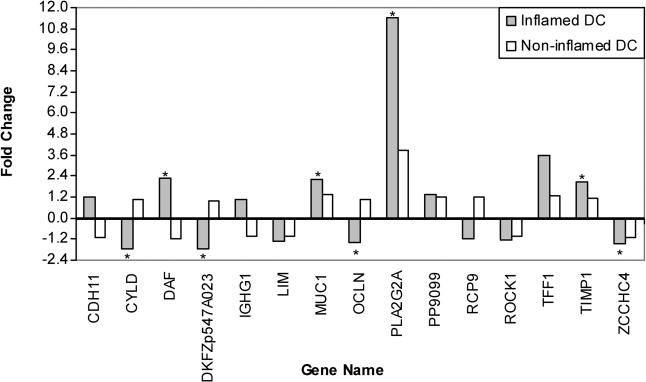
Quantitative Real-Time PCR Results between Normal Controls and Both Non-Inflamed and Inflamed Disease Specificity Controls Quantitative real-time PCR was carried out on individual samples from group 2 patients (including seven or eight inflamed and seven to nine non-inflamed DCs, depending on the availability of cDNA at the time the plates were produced). The DCs include patients with infectious diarrhoea, gastrointestinal inflammation, or irritable bowel syndrome. Patients in this group were not on immunosuppressants or biologicals, but the use of anti-inflammatory drugs was allowed. Results are summarised by a ratio of medians (inflamed DC:normal or non-inflamed DC:normal). Results marked with an asterisk indicate that the real-time PCR result was significant (*p* < 0.05). Complete results, including box-plots and sample numbers analysed in each assay, are included in Figure S1.

### Localisation by Immunohistochemistry

**Figure 4:**
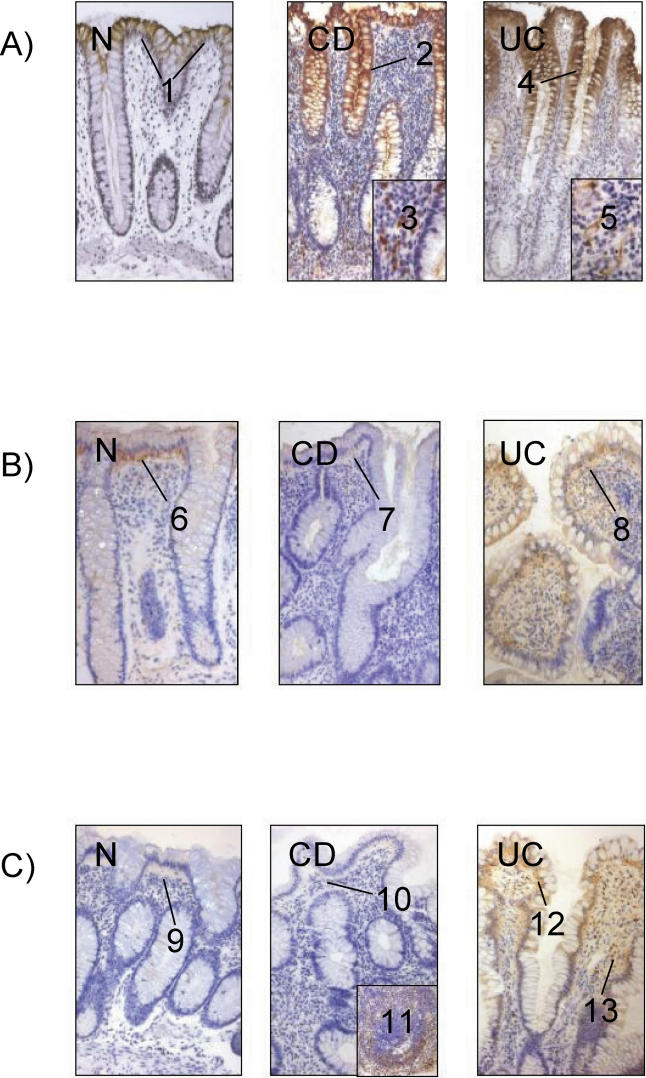
Immunohistochemical Localization of CEACAM1, CSNK1D, and PRKCB1 in Colonic Mucosa Staining of a representative mucosal tissue samples from five normal controls (N), five Crohn disease (CD), and six ulcerative colitis (UC) patients using antibodies against (A) CEACAM1, (B) CSNK1D, and (C) PRKCB1. (A) CEACAM1 immunoreactivity was found in the apical epithelial lining (1), crypts of inflamed tissue (2, 4). Additional staining was detected in immune cells (3) and blood vessels (5). (B) CSNK1D immunoreactivity showed a strong granular staining pattern in normal (6) and the UC group (8), which was located basolaterally. Weaker staining of the apex of the crypts could be detected in CD (7). (C) For PRKCB1, weak staining could be observed in the apical epithelial layer in the normal (9) and CD (10) mucosa and, interestingly, immunoreactivity was found nearly exclusively in the marginal zone of small lymph follicles (11), whereas the lamina propria was immunonegative. In contrast, strong staining was detected in the apical epithelial layer of UC mucosa (12). Furthermore, lamina propria mononuclear cells underlying the epithelial layer were also positive in the UC mucosa (13).

As expression analysis in complex tissues does not allow an identification of the cells responsible for the signal, we used immunohistochemistry to exemplify the approach to genes of interest. Paraffin-embedded sections of samples from the group 1 patients were used. Genes were chosen on the basis that they were formerly not associated with IBD etiopathogenesis and represented a gene in our three main functional groups. Consistent with increased transcript levels measured by microarrays, a marked up-regulation of CEACAM1 protein was seen in IBD when compared to normal controls (Figure 4A). Immunoreactivity was found in the apical epithelial lining in the normal mucosa, whereas it extended down into the epithelial cells of the crypts in the inflamed tissue of CD and UC patients. A staining of vascular structures and mononuclear cells, most likely with a lymphocytic phenotype, was only detected in the lamina propria of inflamed biopsies.

CSNK1D immunohistochemistry staining (Figure 4B) demonstrated expression in the colon, but the modest up-regulation seen in CD in the microarray was not detected by immunohistochemistry. In the biopsies from CD patients, staining of the apex of the crypts could be observed. Immunoreactivity revealed a strong granular staining pattern of intestinal epithelial cells in the normal and UC group, which was located basolaterally.

Staining of PRKCB1 (Figure 4C), that appeared to be down-regulated in the microarray results showed only a weak staining in the apical epithelial layer in the normal and CD mucosa, whereas a stronger staining was present in the UC group. Furthermore, scattered lamina propria mononuclear cells underlying the epithelial layer were also positive. Interestingly, in the CD group, immunoreactivity was found nearly exclusively in the marginal zone of small lymph follicles.

## Discussion

The current understanding of IBD pathogenesis is that of a complex interplay of both genetic and environmental factors that results in an aberrant immune response of the intestinal mucosa. In the past few years, significant progress has been made in elucidating the root causes of CD and UC, with at least three genes now identified in which sequence variations confer disease susceptibility [4–8]. The advent of genomic technologies such as genome-wide microarrays can be used to dissect genes relevant to disease pathophysiology, and allows an unbiased view of both specific and nonspecific (such as response to inflammation) events. In this study we aimed to use the power of genome-wide expression analysis by microarray to dissect novel important regulatory molecules downstream of the multiple primary genetic causes that may initiate the pathophysiologic cascade of inflammation in IBD. Therefore, both specific and nonspecific dysregulation may yield valuable information in identifying new targets for future disease therapy.

In the present study, a total of 650 genes were differentially regulated between normal controls and the two IBD subtypes. More specifically, 500 and 272 differentially regulated transcripts were identified between normal controls and CD and UC patients, respectively. Of note, we observed an imbalance between over- and underexpressed genes in the IBD subtypes. In CD, approximately 84% of differentially expressed genes were found to be down-regulated, compared with 42% of genes in UC. Although this is an interesting finding, it is not prudent at this stage to suggest that broad up- (in UC) or down-regulation (in CD) of genes is a distinctive disease feature, as this finding is highly influenced by types and numbers of genes that are present on any given microarray system. Without exception, none of the 122 differentially expressed genes that were found in both CD and UC, was found to be over expressed in one disease and under expressed in the other. This finding strongly supports the robustness of the observation and the notion of a shared general inflammation profile underlying two clinically divergent forms of IBD, whereas more specific events in the pathophysiologic cascade could be disease-specific.

Disease divergence is also supported by a previous IBD microarray study. Interestingly, when we compared our findings with other CD and UC microarray studies [11–13], we observed few overlaps at the level of individual gene expression (see Table 3), but we did find a high degree of concordance at the level of proposed functional groups and potential mechanisms relevant to IBD (see discussion below). This study therefore not only complements these previous studies, but adds to our knowledge of disease, particularly as the Human UniGene RZPD Set 1 is enriched for as yet unannotated ESTs and, therefore, varies significantly from the commercially available arrays previously used [11,12]. As far as we are aware, the present study is the first to report a systematic assessment of novel unknown genes that might play a role in IBD pathophysiology (44% and 36% of differentially regulated genes identified in CD and UC respectively are, as of yet, not annotated).

The differentiation between CD and UC is a long-debated clinical problem. The large overlap that is seen in pathophysiology is also seen in clinical course. With the exception of gradual differences in activity, all established medical therapies used for colonic inflammation in IBD are applicable to both CD and UC [27]. A considerable percentage of patients appear to change clinical presentation during the course of the disease and are re-diagnosed with one of the other two subforms of IBD. Because of this scenario, the design of the present study did not intend to provide any diagnostic test to differentiate between CD and UC. As a matter of fact, at least one disease gene identified for IBD shows association with both CD and UC [7]. Therefore, the overlap in etiologic factors and pathophysiology between both subforms of IBD could be larger than expected.

Interpreting the functional consequences of changes in gene expression observed in microarray expression screening is one of the major goals of exploratory microarray data analysis. One method used to attempt functional interpretation of microarray data is the use of annotation-based pathway databases such as Gene Ontology (GO). The use of GO does have its limitations, in particular the amount and quality of annotation [28], but it does allow a broad overview of terms to decipher gene pathways. In the present study, we supplemented GO terms, with literature references, and could classify differentially expressed genes into three major groups: immune and inflammatory response; oncogenesis, cell proliferation, and growth; and structure and permeability (see Table 3).

### Immune and Inflammatory Response

Compared to normal mucosa, many genes associated with an aberrant immune response were identified (enrichment significance for this functional group: *p* = 0.00062; category “Immune and Inflammatory Response,” Table 3). Probably more than any other organ in the body, the intestine is a hostile environment, and it is not surprising that a general up-regulation of immune response (enrichment significance *p* = 0.000973) and antigen presentation (enrichment significance *p* = 0.00298) are a common feature of both IBD subtypes, considering the mucosal injury associated with these diseases. In contrast to these shared profiles, leukotriene biosynthesis appears to be more strongly associated with CD (enrichment significance *p* = 0.00483). With the exception of *phospholipase A2, group IIA* (*PLA2G2A),* which has an inferred role in prostaglandin and leukotriene metabolism and was up-regulated in both subtypes, genes associated with leukotriene metabolism, such as *arachidonate 5-lipoxygenase (ALOX5), arachidonate 5-lipoxygenase-activating protein (ALOX5AP), leukotriene B4 receptor 2 (LTB4R2),* and *arginyl aminopeptidase (aminopeptidase B; RNPEP)* were significantly down-regulated in the CD profile (Table 3). In the same vein, the marked down-regulation of CYLD is of interest as this gene has been identified as a key negative regulator of nuclear factor-kappa B (NF-κB) regulation [29–31]. Recent in vitro studies on CYLD regulation have uncovered a novel autoregulatory pathway in which up-regulation of CYLD, due to activation of NF-κB by TNF-α and bacteria, leads in turn to down-regulation of NF-κB signalling [32]. Although real-time analysis showed that this gene was also down-regulated in UC, decreased CYLD expression may represent an inflammation control response that is lost or impaired in some way in IBD. Similarly, inhibition of *NF-κB* signalling can be mediated by *nitrogen oxide (NO)* through the up-regulation of nitrogen oxide synthases (encoded by *NOS2A,* Table 3), which produces NO from arginine, along with other genes associated with proline and arginine metabolism, such as proline 4-hydroxylase (*P4HB,* Table 3) and aldehyde dehydrogenase 2 (*ALDH2,* Table 3), suggests over stimulation of this metabolic pathways in the UC profile. It is worth noting that it is not clear whether NO is deleterious or beneficial in gastrointestinal disease [33]. However, considering the well-documented potent inflammatory role NF-κB activation has in IBD [29–31], further examination of this complex issue is well worthwhile.

Finally, within this gene category, expression of *CEACAM1* (a known neutrophil activation marker and regulator of T cell function [34,35]) was examined by immunohistochemistry. We have shown that this protein is present in blood or lymphatic vessels of the inflamed mucosa (Figure 4A, insets), consistent with its role in angiogenesis [36]. Infiltrating immune cells in the lamina propria were stained by anti-CEACAM1 antibody in CD, but not in UC, suggesting that CEACAM1 may be a better therapeutic target in CD. Expression of this adhesion molecule was distributed along the surface of the normal and IBD mucosa (Figure 4A), but distribution tended to more deeply penetrate the crypts in the IBD mucosa. It is tempting to speculate that this finding may have a role to play in increasing bacterial load in IBD, since CEACAM1 can act as a receptor for certain bacteria [37]. However, association of immunohistochemical findings with IBD pathogenesis are strictly speculative, and further studies, outside the scope of the present study, would need to be carried out to establish their exact role in disease.

### Oncogenesis, Cell Proliferation, and Growth

Within this category, an enrichment of genes associated with cell growth and proliferation was found for genes up-regulated in UC (enrichment *p* = 0.0404; category “Oncogenesis, Cell Proliferation, and Growth,” Table 3)**.** This finding is similar to results from previous microarray studies, which reported involvement of cancer-related genes in IBD [11,12], although we report mostly different genes. During chronic inflammation, the constant repair mechanisms require precise controlling of molecular remodelling, cell proliferation, and growth [38], and dysregulation of any of these processes is followed by abnormalities that can lead to development of cancer [39], which is one of the main long-term complications of UC. Interestingly, one of the uncharacterised transcripts (Chromosome 14 ORF 125/Mm, Rn, GenBank accession number AL117511; see Table 4) was assigned as potentially involved in regulation of apoptosis, which concurs with the above-mentioned studies. Significant up-regulation of cancer-related genes in the UC profile is potentially important, considering reports of increased risks of developing colorectal carcinoma in this disease [40,41]. Up-regulated genes in the UC profile included *v-myb myeloblastosis viral oncogene homolog 2* (*MYBL2,* Table 3) and several members of the S100 protein family, whose expression has been reported to promote cell proliferation and are linked with increased malignancy or tumour progression in colon cancer [42–45]. As confirmed by real-time PCR, TFF1 expression was also markedly higher in the UC group. This finding may be very interesting considering earlier studies describing increased TFF1 protein in colorectal cancer [46] and cancer development in TFF1 knock-out mice [47]. The potentially specific role this gene may have in UC is emphasized by the fact that we find no evidence of dysregulation of this gene in either CD or disease specificity controls. Similarly, the Wnt pathway is of intense interest, as aberrant activation is a common signalling abnormality in human cancers [36,37]. As members of the *casein kinase I (CKI)* gene family have been implicated in the regulation of Wnt-targeted gene expression [48,49], we used immunohistochemical analysis to localise the expression of this gene in IBD. Although contrary to the array results (where this gene was up-regulated in CD but not UC), the novel immunohistochemical finding of basolateral staining of CSNK1D in normal and UC epithelial cells, but only faint staining in the crypts of CD, may imply a role for Wnt signalling in IBD pathogenesis. This possibility would need to be investigated by more in-depth functional studies. The divergent expression patterns of genes involved in this category might yet prove useful in understanding the different risks of developing colorectal carcinoma between the two IBD subtypes [50].

### Structure and Permeability

Constant tissue damage and injury of the intestinal surface are part of the pathophysiologic mechanisms in chronic disorders such as IBD that require continuous repair of the epithelium [38]. Enhanced permeability for inert macromolecules is a well-described clinical feature of CD [51]. Most interestingly, *DLG5,* a recently discovered disease gene in IBD, also appears to be involved in mechanical integrity of epithelial barriers [7,52,53]. The organic cation transporter genes *SLC22A4* and *SLC22A5,* which are expressed in epithelial cells, have also been identified recently as disease genes for CD [8]. There is an enrichment of genes associated with structure and permeability in this study (enrichment *p* = 0.0309; category “Structure and Permeability,” Table 3) as well as in previous microarray studies [11–13]. Several genes in this category were ubiquitously regulated in both IBD and non-IBD samples, reflecting known gene dysregulation in disease where inflammation and wound healing are recurrent events. These include paracellular permeability (down-regulation of *OCLN*) [54], degradation of extracellular matrix (up-regulation of *TIMP* and *MMP2*) [55,56] and barrier protection against bacterial invasion of the epithelial surface (up-regulation of *MUC1*) [57]. Conversely, several genes *(LIM* and *ROCK)* appear specifically relevant to disease processes in IBD, as no difference in regulation was observed in inflamed non-IBD patients by real-time PCR. Members of the cadherin superfamily, integral membrane proteins that mediate calcium-dependent cell-cell adhesion, have been shown to be involved in epithelial cell migration and resealing the site of tissue damage in the intestinal mucosa [58]. In this study, we show shared up-regulation of *cadherin 11 (CDH11)* in both IBD subtypes, too, but not in inflamed non-IBD tissue. It is tempting to speculate that this member of the cadherin family could also be involved in restructuring processes in the intestinal mucosa. In the same context, we identified two uncharacterised transcripts (GenBank accession numbers N48794 and AF087994) as differentially regulated, which were assigned a role in cell-cell adhesion (one of them containing cadherin repeats).

Cell migration in response to wound healing is regulated in part by cell adhesion processes, such as Rho-ROCK-mediated cytoskeletal reorganization [59,60]. Interestingly, members of this pathway, *ROCK1* and *LIM*, were down-regulated in both IBD subtypes in our PCR experiments (but not in inflamed DCs), which might indicate a potential decline in cell migration and an impaired ability to maintain epithelial integrity in IBD. Furthermore, immunohistochemistry staining of *PRKCB1*, which has been shown to interact with *LIM* [59], showed that lamina propria cells were preferentially stained in UC, but not CD or normal controls, possibly indicating a unique role for this protein in UC. Interestingly, a genetic variant in *DLG5,* which was recently associated with IBD [7], is a PDZ-containing scaffolding protein that associates with β-catenin and is a binding partner of vinexin at cell-cell contacts [52]. *LIM*, in addition to its signalling function by interacting with *PRKCB1* through its *LIM* domain [59], also contains PDZ domains, which may be used as sites for interaction of scaffolding proteins. These findings might represent interesting targets for further functional characterization in the context of wound healing and regeneration.

This study represents the largest sample number used in IBD microarray studies to date and focuses on three groups, namely normal control, CD, and UC. It would be of interest to substratify the groups according to other factors, which may then reveal factor-specific effects. For example, heterogeneity in the age distribution of the patient cohort could have the potential to introduce a bias. To address age-related effects, an independent analysis omitting age outliers from the analysis was performed. This did not result in significant changes in the presented list of differentially expressed genes, with only one out of 650 genes *(Zyxin)* failing to meet the cutoff criteria. Similarly, some patients, although meeting both clinical and endoscopic study inclusion criteria, had no acute infiltrate in their histology assessment (an “A0” score on histology). The lack of a reliable histological score reflecting disease activity is a well-known phenomenon in clinical research in IBD [27]. Elimination of individuals with an “A0” histology score did not change the results in a de novo analysis. We therefore conclude that the presented cohort composition was appropriately selected to identify differentially expressed genes in CD and UC, while noting that higher numbers of microarray samples would be necessary to sufficiently delineate the effects of additional clinical parameters. A future agenda would be the assessment of differential gene expression according to multiple subphenotypes and natural disease course. A prospective cohort that is adequately powered in size has been assembled and is currently under investigation.

In conclusion, this study reports our gene findings using a large, whole-genome filter system (Human UniGene set RZPD 1). Our results indicate that there are differences in the gene expression patterns between normal colonic mucosa and both CD and UC. The main findings point to novel, important molecules in abnormal immune regulation and the highly disturbed cell biology of the intestine in IBD pathogenesis. We suggest a host of novel genes that could be implicated in IBD pathophysiology and pathogenesis. The large number of involved mechanisms underlines the complexity of the IBD phenotype, but offers a large number of potential starting points for the development of new therapeutic strategies in the management of IBD.

## Supporting Information

Dataset S1 Expression values and GO annotations for 650 sequence-verified genes discussed in the manuscript (409 KB TXT).

Figure S1 Boxplot Results of Real-Time PCR Experiments on 15 Genes Real-time PCR was carried out for 15 genes as described in main text and shown in Figures 2 and 3. The number of samples per analysis is given in parentheses for each group analysed: normal controls; CD; UC; DC, actively inflamed (Inflamed_DC); and DC, not actively inflamed (Non-inflamed_DC). Graphs are provided for: *CDH11* (1a), *CYLD* (1b), *DAF* (1c), *DKFZp547A023* (1d), *IGHG1* (1e), *LIM* (1f), *MUC1* (1g), *OCLN* (1h), *PLA2G2A* (1i), *PP9099* (1j), *RCP9* (1k), *ROCK1* (1l), *TFF1* (1m), *TIMP1* (1n), and *ZCCHC4* (1o).
(78 KB PPT).

Table S1 Real-Time TaqMan Probe and Primer Sequences (131 KB PDF).

Table S2 Differentially Regulated Genes in IBD Linkage Regions Identified on Microarrays (131 KB PDF).

### Accession Numbers

The entire dataset discussed in this paper is compliant to the Minimum information about a microarray experiment (MIAME) criteria and is deposited at Gene Expression Omnibus (http://www.ncbi.nlm.nih.gov/projects/geo/) under the series accession number GSE1710 (platform ID, GPL284; dataset IDs, GSM29595–GSM29625).

**Patient Summary**

**Background**

Crohn's disease and ulcerative colitis are both inflammatory diseases of the gut, and there is a great deal of overlap between them in the symptoms that patients have. However, both diseases also have distinctly different clinical features that allow, in about 80% of patients, a differentiation between the two diseases. Although some genes have been shown to be associated with one or the other type of the disease, the cascade of events that leads to the symptoms we call disease is not fully understood.

**Why Was This Study Done?**

New technology allows researchers to compare the function of many thousands of genes side by side. These researchers wanted to look at how the genes that are expressed (active) differ between samples taken from the guts of people with these two conditions.

**What Did the Researchers Do and Find?**

They compared gut samples from people with inflammatory bowel disease with others taken from people with normal colon, and others with unrelated colonic diseases. They found differences in the pattern of expression of hundreds of genes between Crohn's disease and ulcerative colitis and samples from other diseases and normal colonic mucosa. The types of genes mostly altered were those involved in immunity (how the body protects itself against outside substances); those involved in mechanisms regulating how cells multiply (which can go wrong in cancer—one of the complications of ulcerative colitis) and those that control how the cells of the gut remain intact.

**What Do These Findings Mean?**

They point to some new areas of research for these diseases, and possible targets for new drugs. They also indicate how complex these two diseases are, and suggest that there is not just one trigger for the diseases.

**Where Can I Get More Information Online?**

National Institute of Diabetes and Digestive and Kidney Diseases, National Institutes of Health has an alphabetical list of digestive diseases and links to clinical trials in this area: http://digestive.niddk.nih.gov/ddiseases/

American and the German Crohn's and Colitis foundations:

http://www.ccfa.org; http://www.dccv.de

Kompetenznetz—Darmerkrankungen (in German):

http://www.kompetenznetz-ced.de

The researchers' home page:

http://www.ikmb.uni-kiel.de

## Abbreviations

5-ASA: 5-aminosalicylic acid
CAI: colitis activity index
CD: Crohn disease
CDAI: Crohn disease activity index
DC: disease specificity control
EST: expressed sequence tag
F: female(s)
GO: Gene Ontology
IBD: inflammatory bowel disease
IL-8: *interleukin-8*
NF-κB: *nuclear factor-kappa B*
NO: nitrogen oxide
ORF: open reading frame
TNF-α: *tumour necrosis factor-alpha*
UC: ulcerative colitis
