# Supplementary material for: Dissection of the Inflammatory Bowel Disease Transcriptome Using Genome-Wide cDNA Microarrays
Source: PLoS Med. 2005 Aug 23;2(8):e199. doi: 10.1371/journal.pmed.0020199 (PMC1188246; doi:10.1371/journal.pmed.0020199)
Supplement: Supplementary file 1 [file pmed.0020199.st001.pdf]

Supplemental Data

Supplemental table 1: Real-time TaqMan probe and primer sequences

Transcript	*Forward (5' to 3')*	*Reverse (5' to 3')*	*Probe or context sequence* (5' to 3')*
β-actin	AGCCTCGCCTTTGCCGA	CTGGTGCCTGGGGCG	CCGCCGCCCGTCCACACCCGCC
IL-8	GGAATTGAATGGGTTTGCTAGAA	TGTGGATCCTGGCTAGCAGAC	TGGAAATCCTGGATTTTTTTCTGTTAAATCTGGCAA
TNFα	AGGCGGTGCTTGTTCCTCA	GTTCGAGAAGATGATCTGACTGCC	CCAGAGGGAAGAGTTCCCCAGGGAC
CDH11	ACCAACACCCTCACCATCAAA	GGCCGGCGTTCAGAATG	TGCGGGTGCGACGTGAACG
DAF	CGAGGTGCCAACAAGGCTAA	CCTGGACGGCACTCATATTCC	TTCTGCATCCCTCAAACAGCCTTATATCACTCT
IGHG1	GCCCCAGTTGCTGGC	AGCTGGGTCCCGCTGC	TTTCAATCCAAGCATAACTCAGTGACGCAT
MUC1	GTGCCCCCTAGCAGTACCG	GAAGTGGCTGCCACTGCTG	TGAGAAGGTTTCTGCAGGTAATGGTGGCA
TFF1	GACACCTCAGACACGCTTCT	GAGTAGTCAAAGTCAGAGCAGTCAA	CCTCGGCTCACAACAC
TIMP1	TTCTGGCATCCTGTTGTTGCT	TGACGAGGTCGGAATTGCA	CCCCCAGCAGGGCCTGCACC
CYLD*			TCGGGATGGTGGTCAGAATGGCTTC
DKFZp547A023*			AGGCTGACTCAACAGTTGGAATTTG
LIM*			AAAATGGCCCACCAAGAAAACACAT
OCLN*			CCACGCCGGTTCCTGAAGTGGTTCA
PLA2G2A*			AGCAGAATCACCTGTGCAAAACAGG
PP1628*			GAACAAGGTCAGCGACATCAAATTC
ROCK1*			AATTGGATGAAGAGGGAAATCAAAG
RCP9*			GGAGATCCAGCTGATGGTGGAAGAG
ZCCHC4*			GCACATCCAAGGATGGCAGGAAATG

Probes and primers were either designed to non-redundant sequence using Primer Express V2.0 (Applied Biosystems), or ordered from Applied Biosystems as Assays-on-Demand (AoD). * AoD gene assays are provided with context sequence surrounding the assay location.
