# Supplementary material for: Dissection of the Inflammatory Bowel Disease Transcriptome Using Genome-Wide cDNA Microarrays
Source: PLoS Med. 2005 Aug 23;2(8):e199. doi: 10.1371/journal.pmed.0020199 (PMC1188246; doi:10.1371/journal.pmed.0020199)
Supplement: Supplementary file 2 [file pmed.0020199.st002.pdf]

Supplemental Table 2: Differentially regulated genes in IBD linkage regions identified on microarrays

IBD locus / gene name	*Symbol*	*Cytoband*	*NC vs CD*	*NC vs UC*	*Hypotheses for possible involvement in IBD*		
			Fold-change	*Fold-change*	*Immune and inflammatory response*	*Cell growth and proliferation*	*Structure and permeability*
IBD 1							
Matrix metalloproteinase 2	MMP2	16q12.2	1.98	1.8			x
Solute carrier family 6 (neurotransmitter transporter, noradrenalin), member 2	SLC6A2	16q12.2	-1.63				x
Cylindromatosis (turban tumor syndrome)	CYLD	16q12-q13	-1.53		x	x	
IBD 2							
Protein kinase, lysine deficient 1	PRKWNK1	12p13.3	1.37				
KIAA1238 protein	KIAA1238	12p13.31	-1.36				
Retinoic acid induced 3	RAI3	12p13-p12.3		1.44			
CD63 antigen (melanoma 1 antigen)	CD63	12q12-q13		1.58	x		
Poly(rc) binding protein 2	PCBP2	12q13.12-q13.13		1.25			
Tachykinin 3 (neuromedin K, neurokinin beta)	TAC3	12q13-q21	-1.6				
Ubiquitin specific protease 15	USP15	12q14	-1.45				
Dual-specificity tyrosine-(Y)-phosphorylation regulated kinase 2	DYRK2	12q14.3	-1.36				
Protein kinase, AMP-activated, beta 1 non-catalytic subunit	PRKAB1	12q24.1	1.32				
KIAA1033 protein	KIAA1033	12q24.11	-1.34				
Activin A receptor type II-like 1	ACVRL1	12q11-q14	1.35	1.43			
IBD 3							
Translocation associated membrane protein 2	TRAM2	6p21.1-p12	-1.46				
Cyclin-dependent kinase inhibitor 1A (p21, Cip1)	CDKN1A	6p21.2	-1.46				
B-factor, properdin	BF	6p21.3		2.37			
Flotillin 1	FLOT1	6p21.3		1.47			
Major histocompatibility complex, class I, A	HLA-A	6p21.3	2.17		x		
Major histocompatibility complex, class II, DQ alpha 1	HLA-DQA1	6p21.3	-1.47		x		
MHC class I polypeptide-related sequence A	MICA	6p21.3	-1.4		x		
Sialidase 1 (lysosomal sialidase)	NEU1	6p21.3	-1.28				x
Chromosome 6 open reading frame 69	C6orf69	6p21.31	-1.44				
Zinc finger protein 305	ZNF305	6p22.2-p21.3	-1.38				
Colipase, pancreatic	CLPS	6pter-p21.1	-1.48				
Major histocompatibility complex, class I, B	HLA-B	6p21.3	1.96	2.19	x		
Major histocompatibility complex, class II, DR beta 3	HLA-DRB3	6p21.3	1.71	2.09	x		
Immediate early response 3	IER3	6p21.3	1.95	2.31	x		
IBD 4							
Suppressor of Ty 16 homolog (S. Cerevisiae)	SUPT16H	14q11.2	-1.45				
Leukotriene B4 receptor 2	LTB4R2	14q11.2-q12	-1.59		x		
Adaptor-related protein complex 1, gamma 2 subunit	AP1G2	14q11.2	-1.44	-1.41			
Chromosome 14 open reading frame 125	C14orf125	14q12	-1.33	-1.25			
IBD 5							
Protein phosphatase 2 (formerly 2A), catalytic subunit, alpha isoform	PPP2CA	5q23-q31	-1.48				
H2A histone family, member Y	H2AFY	5q31.3-q32	-1.47				
Secreted protein, acidic, cysteine-rich (osteonectin)	SPARC	5q31.3-q32	1.47				
Solute carrier family 26 (sulfate transporter), member 2	SLC26A2	5q31-q34		-1.37			x
Purine-rich element binding protein A	PURA	5q31	-1.4	-1.3		x	
IBD 6							
RAB8A, member RAS oncogene family	RAB8A	19p13.1		1.41		x	
A kinase (PRKA) anchor protein 8-like	AKAP8L	19p13.13-p13.12		1.55			
Jun B proto-oncogene	JUNB	19p13.2	1.52	1.62		x	
IBD 7							
Eyes absent homolog 3 (Drosophila)	EYA3	1p36		1.37			
Chromosome condensation 1	CHC1	1p36.1	-1.34				
H. s. transcribed seq. with similarity to prot. ref:NP_060312.1 hyp. prot. FLJ20489	-	1p36.1	-1.54	-1.49			
Hypothetical gene supported by AK124869	LOC400745	1p36.12	-1.37				
KIAA1922 protein	KIAA1922	1p36.13	-1.32				
Kinesin family member 1B	KIF1B	1p36.2	1.42				
Vacuolar protein sorting 13D (yeast)	VPS13D	1p36.21	-1.57				
Hypothetical protein MGC26818	MGC26818	1p36.32		1.7			
Proline-rich nuclear receptor coactivator 2	PNRC2	1p36.11	1.35	1.37			
Putative nfkb activating protein	KIAA0720	1p36.23	-1.25	-1.23	x		
Enolase 1, (alpha)	ENO1	1p36.3-p36.2	1.47	1.37			
IBD 8							
KIAA0296 gene product	KIAA0296	16p11.2		1.3			
Protein kinase C, beta 1	PRKCB1	16p11.2	-1.5				x
Hypothetical protein LOC146174	LOC146174	16p12.3	2.6				
Poly(A)-specific ribonuclease (deadenylation nuclease)	PARN	16p13		1.32			
Kruppel-like zinc finger protein GLIS2	GLIS2	16p13.3		2.4			
Hemoglobin, alpha 2	HBA2	16p13.3		1.29			
Ubiquitin-conjugating enzyme E2I (UBC9 homolog, yeast)	UBE2I	16p13.3		1.5			
IBD 9							
Basic helix-loop-helix domain containing, class B, 2	BHLHB2	3p26		1.3			

IBD locus is the classification according to OMIM and only those genes in IBD1–IBD9 are listed in this table.
NC: Normal Control; CD: Crohn's Disease, UC: Ulcerative Colitis: Fold changes are only listed if p-value <0.0015
